# Supplementary figures and images for: Tissue-Specific Prevalence and Clonal Architecture of BRCA1/2 LOH-Inducing Chromosomal Aneuploidy
Source: bioRxiv. 2026 Apr 21:2026.04.17.718766. Preprint. [Version 1] doi: 10.64898/2026.04.17.718766 (PMC13131466; doi:10.64898/2026.04.17.718766)

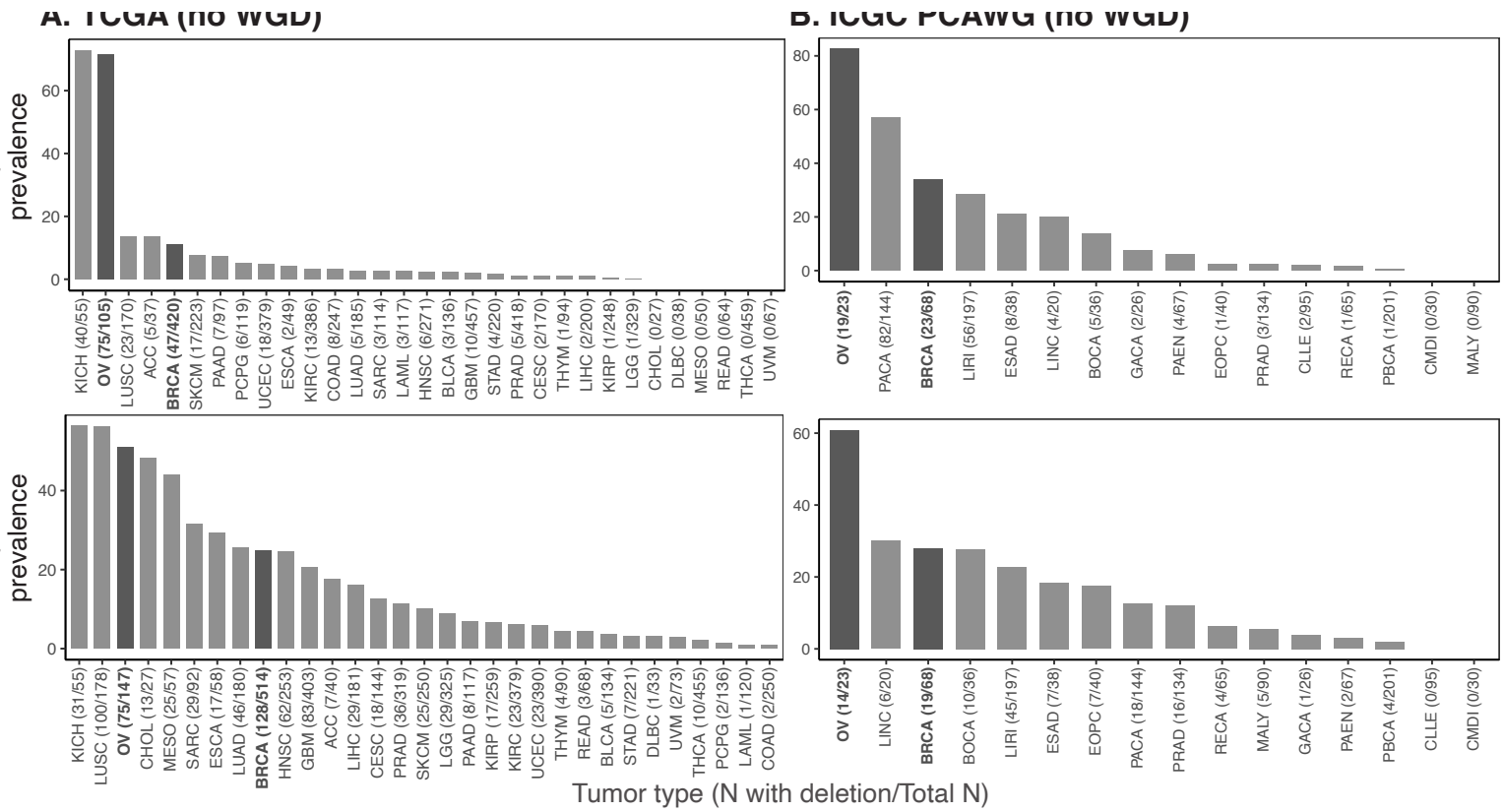

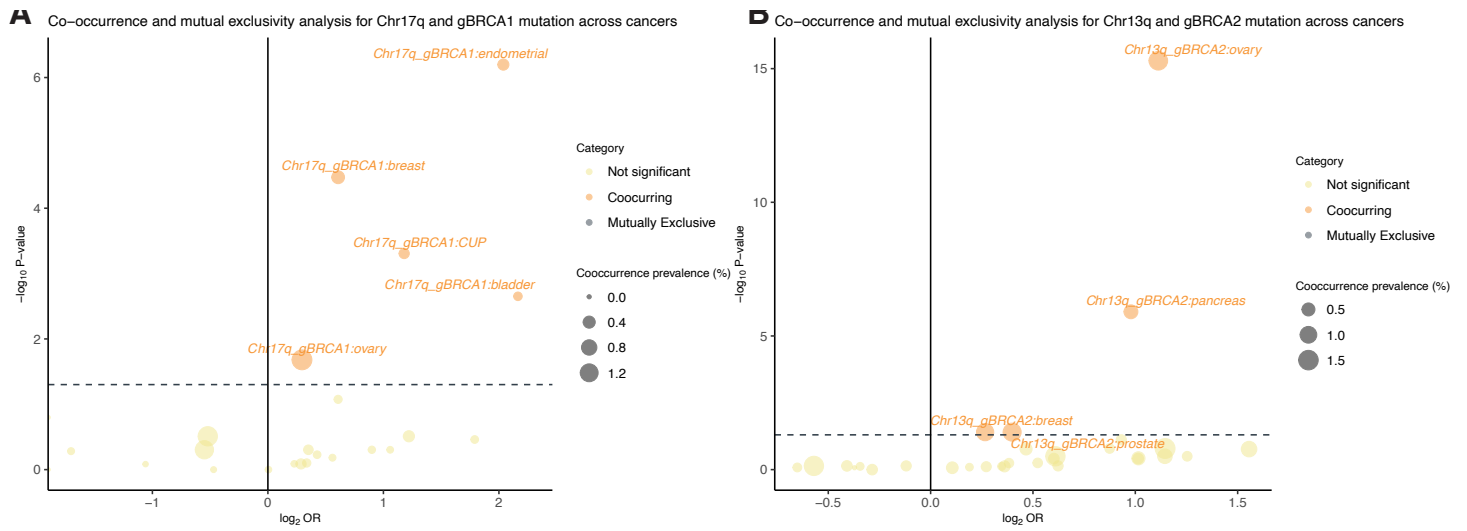

**A.**

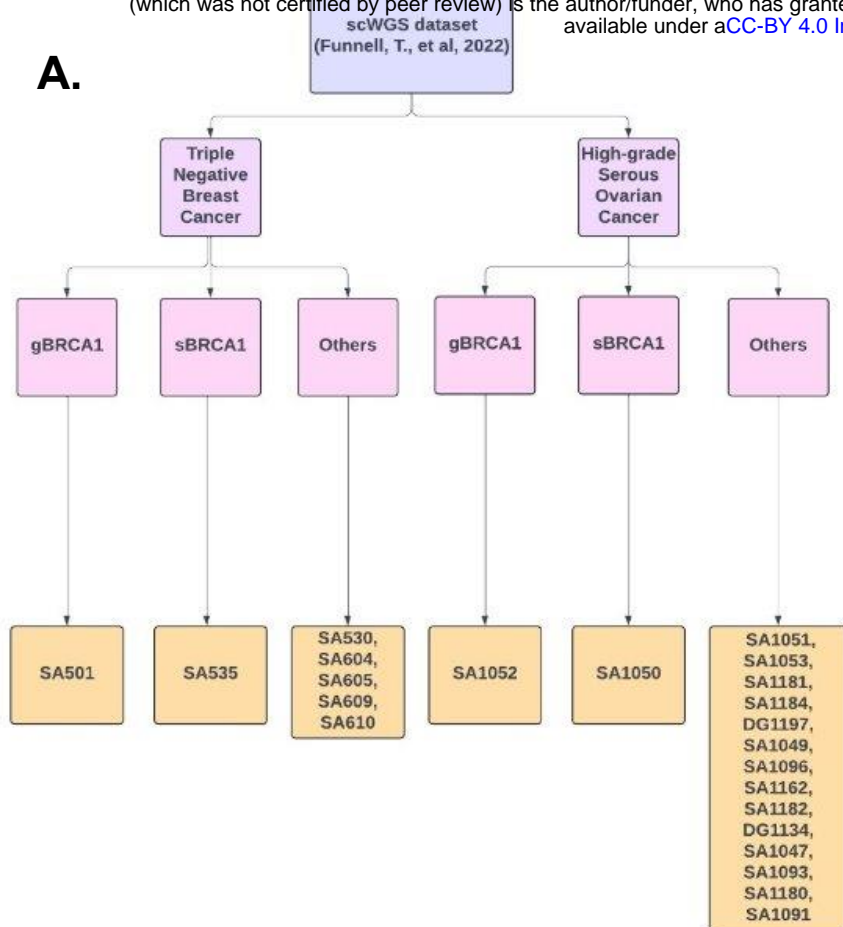

**B.**

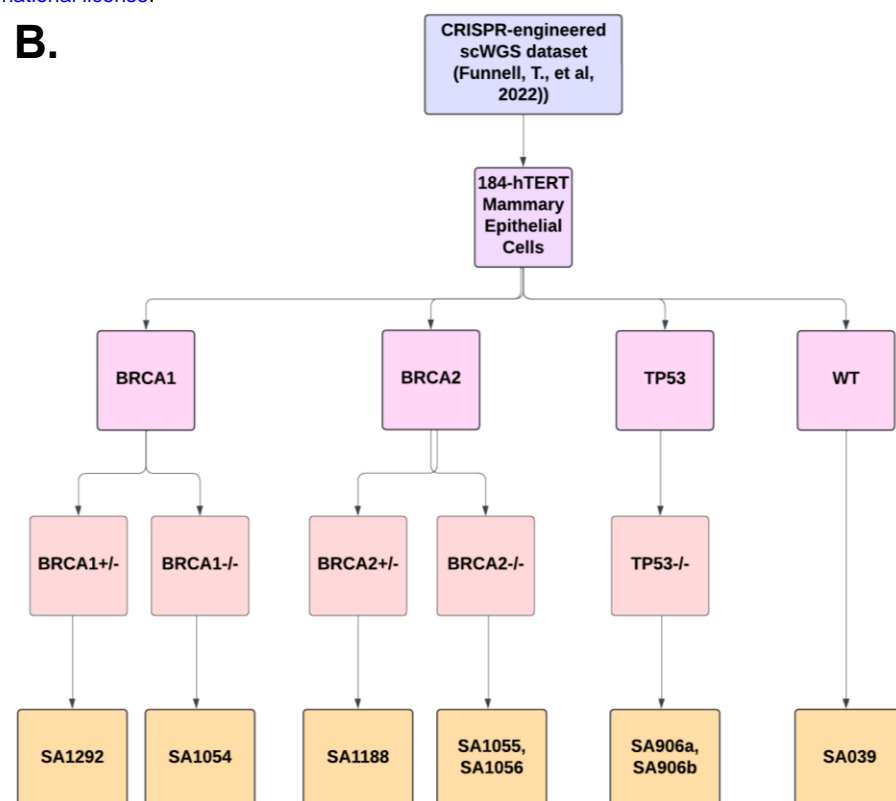

Supplement: 1 — Supplementary Figure 1. The prevalence of chr17q and chr13q deletions across tumor types in non-WGD samples of the (A) TCGA and (B) ICGC PCAWG cohorts. The x-axis lists the tumor types along with the number of samples with deletions out of the total number of samples analyzed, e.g., “CANCER (#N affected/#Total N)”. The y-axis indicates the percentage of samples with deletions for each tumor type. Tumor types with a higher prevalence of deletions are shown on the left, decreasing towards the right. Supplementary Figure 2. Co-occurrence and mutual-exclusivity analyses using FCore cohort to evaluate the statistical associations between (A) chr17q deletion and gBRCA1 and (B) chr13q deletion and gBRCA2. Supplementary Figure 3. Single cell analysis of copy number deletions of chromosome arms 17q and 13q in gBRCA1/2 vs non-carrier tumor samples and genetically engineered cells. (A) Summary flowchart of single-cell datasets and samples used in analyses. The left panel displays data from Funnell, T. et al. (2022), including scWGS-analyzed both TNBC and HGSC cells possessing gBRCA1, sBRCA1 and WT BRCA1 variants. The right panel shows data from William et al. (2024). Percentage of inferred single-cell copy number deletions on chromosome arms (B) 17q and (C) 13q in TNBC samples inferred from scRNA-seq data. Plots compare the percentage of CNDs between gBRCA1 tumor cells and non-carrier tumor cells. [file NIHPP2026.04.17.718766V1-supplement-1.pdf]
